# Optimization of Sensor Network for Velocity-Free Acoustic Emission Source Localization in Construction Materials

**DOI:** 10.3390/ma19112399

**Published:** 2026-06-04

**Authors:** Xiaofeng Huang, Yang Liu, Longbin Yang, Longjun Dong

**Affiliations:** 1 School of Resources and Safety Engineering, Central South University, Changsha 410083, China; 195502037@csu.edu.cn (X.H.); lj.dong@csu.edu.cn (L.D.); 2 Woxi Gold-Antimony Mine, Hunan Chenzhou Mining Co., Ltd., Huaihua 419605, China; 3 School of Resources Environment and Safety Engineering, University of South China, Hengyang 421001, China; 20252002210144@stu.usc.edu.cn

**Keywords:** acoustic emission, sensor network optimization, source localization, pencil-lead break test, non-destructive testing, construction material

## Abstract

Acoustic emission (AE) source localization provides important spatial information for damage characterization and fracture evolution analysis in construction materials, while its accuracy and applicability are strongly dependent on sensor network design. This study proposes an optimization framework for selecting an effective six-sensor network for velocity-free AE source localization in construction materials. The source coordinates are determined by solving a nonlinear inverse problem using the Levenberg–Marquardt algorithm, and candidate sensor subsets are evaluated by combining location error metrics with the number of effective localization results to quantify the effective monitoring range for damage characterization. The framework is investigated through numerical simulations and pencil-lead break tests on a 600 mm × 600 mm ceramic tile. Among different six-sensor configurations, the best-performing layouts place four sensors at the outer corners and two sensors at the horizontal or vertical inner corners. A benchmark comparison with the Fisher-information-based optimized sensor network and sensitivity analyses further show that the optimized sensor network maintains higher effective monitoring ranges under arrival-time noise, velocity uncertainty, and sensor coordinate perturbations. The proposed approach provides a useful reference for robust and cost-effective AE sensor network design in damage monitoring, fracture characterization, and nondestructive evaluation of construction materials.

## 1. Introduction

Acoustic emission (AE) monitoring is widely used as a nondestructive technique for damage localization [1,2,3], damage characterization [4,5,6], and failure assessment under mechanical loading [7,8]. Among different AE analysis tasks, source localization is particularly important because the spatial coordinates of AE events provide a basis for identifying cracking zones, tracking fracture development, and interpreting damage patterns in construction materials. However, the accuracy and robustness of AE source localization are often affected by non-ideal wave propagation conditions, material heterogeneity, and limitations in sensor placement. These factors may lead to considerable location errors, especially near boundary regions or under constrained sensor layouts. Therefore, improving the accuracy and stability of AE source localization remains essential for reliable damage characterization and fracture assessment in construction materials.

Localization performance is governed by multiple factors, including the choice of optimization algorithm, uncertainties in arrival-time picking, the treatment of wave velocity, sensor network geometry, and environmental variations such as temperature [9,10,11]. Classical derivative-based methods, such as Geiger’s method and weighted least squares, can be computationally efficient but may converge to local minima depending on initialization and data quality [12,13]. In contrast, derivative-free or global-search strategies, such as particle swarm optimization and simulated annealing, are less sensitive to initialization but typically incur higher computational cost [14,15]. Comparative studies have suggested that no single solver is uniformly best across scenarios and that hybrid strategies may be advantageous when the misfit landscape is highly nonconvex [16]. This trade-off between accuracy, stability, and computational expense is one of the practical tensions in AE localization.

Arrival-time uncertainties are another dominant error source. Standard picking strategies, such as the short-term average/long-term average (STA/LTA) ratio, can be sensitive to parameter choices and noise characteristics [17], motivating alternative criteria such as the Akaike information criterion and subsequent refinements [18]. More recent developments incorporate cross-correlation, wavelet-based processing, image-connectivity approaches, improved energy-ratio criteria, and machine-learning-based pickers [19,20,21,22,23]. These advances reduce picking errors, yet the remaining uncertainty still propagates through the inverse problem and interacts with sensor geometry, implying that algorithmic improvements alone do not eliminate layout-dependent performance degradation.

A further challenge is the treatment of wave velocity. Many traditional approaches assume a constant velocity, effectively modeling the medium as homogeneous and isotropic. In practice, velocity can vary due to structural heterogeneity, voids, boundary conditions, and environmental factors, which introduce model mismatch and systematic localization bias. Li et al. [24] investigated the influence of pre-measured velocity on location accuracy. They found that the location error in a 300 mm × 300 mm sensor network is up to 87 mm with a velocity error of 200 m/s. Therefore, even a small velocity error will lead to a large decrease in the accuracy and stability of source localization. To mitigate this issue, velocity-free formulations treat the velocity as an unknown or avoid prescribing it explicitly, improving robustness in isotropic or weakly anisotropic settings [25,26]. For more anisotropic cases, delta-T mapping and its automated variants have been proposed, but they require either extensive calibration experiments or synthetic database generation, which can be costly or time-consuming depending on the application [27,28,29]. This motivates studying sensor networks for velocity-free localization, which mitigates model mismatches due to a prescribed velocity but remains sensitive to sensor geometry.

Sensor network optimization has a long history in related fields such as seismology, where Monte Carlo strategies and geometric criteria have been used to design monitoring networks [30,31,32]. Subsequent studies evaluated the effects of station density, geometry, and array integration on location uncertainty [33], while other work derived geometric criteria associated with spreading and directional control [34]. In the AE field, the influences of array configuration, sensor number, and sensor spacing have also been analyzed [35]. For example, Yin et al. [36] proposed Z-shaped sensor clusters for acoustic source localization in anisotropic plates, demonstrating that sensor arrangement can improve localization performance under direction-dependent wave propagation. Tayfur et al. [37] developed a cluster-based sensor selection framework for AE source localization in concrete, showing that appropriate sensor selection can improve localization efficiency and accuracy. Dong et al. [38] analyzed the error distribution and influencing factors of AE source localization for rectangular sensor networks, indicating that localization errors are strongly affected by source position and network geometry. Rui et al. [39] further optimized MS/AE monitoring sensor networks using Fisher information and an improved encoding framework, providing a quantitative basis for network design. In addition, Zhao et al. [40] proposed a triangular AE source location method considering anisotropic propagation behavior, further confirming the importance of sensor layout and propagation characteristics in localization accuracy. More recently, Liu et al. [41] combined deep learning and AE technology to localize damage in in-service reinforced concrete columns and showed that localization performance and monitoring capability were strongly dependent on sensor layout, with different arrangements being preferable for shallow and deep damage regions. These studies indicate that the effectiveness of a given sensor layout depends not only on the localization model itself, but also on how and where the error is assessed. Evaluations restricted to sources inside the sensor network may not reflect behavior near boundaries or in exterior corner regions. This consideration motivates assessing sensor configurations over both interior and exterior regions when designing layouts for AE localization in construction materials.

In general, previous studies on sensor network optimization have mainly focused on velocity-based localization and have evaluated localization accuracy primarily for sources located inside the sensor network. In this study, we investigate six-sensor layouts for velocity-free AE source localization in construction materials using both numerical simulations and pencil-lead break (PLB) tests on a ceramic tile. To enable a unified comparison of candidate layouts, the number of effective localization results (NELR) is introduced as an evaluation index to quantify the effective monitoring range. Furthermore, to better position the proposed method with respect to existing approaches, the optimized sensor network obtained based on Fisher information and improved encoding framework (SN-FIIEF) [39] is selected as a benchmark for comparison. By comparing localization performance in both numerical simulations and PLB tests, the effectiveness and potential advantages of the proposed sensor layout strategy can be more clearly demonstrated.

## 2. Materials and Methods

### 2.1. Velocity-Free Localization Model

To reduce the influence of velocity errors, the source positions in numerical simulations and PLB tests were determined using the velocity-free localization method. The P-wave velocity is unknown and represented by v. The coordinates of the source and the sensors are, respectively, represented by $\left(x_{0},y_{0}\right)$ and $S_{i}(x_{i},y_{i})$ where i=1,2,3,4…,n and n is the total number of sensors. The arrival time recorded by the sensor $S_{i}$ can be calculated by

(1) $$t_{i}=\frac{R_{i}}{v}+t_{0},$$

where $R_{i}$ is the distance between the sensor $S_{i}$ and the source, and $t_{0}$ is the offset time of the source. According to the distance formula, one has

(2) $$R_{i}=\sqrt{{\left(x_{i}-x_{0}\right)}^{2}+{\left(y_{i}-y_{0}\right)}^{2}}.$$

The time difference in arrivals (TDOA) between sensor $S_{i}$ and $S_{j}$ are calculated by

(3) $$∆t_{ij}=t_{i}-t_{j}=\frac{R_{i}-R_{j}}{v}.$$

The degree of deviation between the observed TDOA $\Delta t_{ij}^{obs}$ and the calculated TDOA $\Delta t_{ij}^{cal}$ represents the deviation between the potential solution $\left(x^{\prime },y^{\prime },v^{\prime }\right)$ and the real solution $\left(x_{0},y_{0},v\right)$, which is described by

(4) $$D\left(x^{\prime },y^{\prime },v^{\prime }\right)=\sum_{i=1}^{n}{\left(∆t_{ij}^{obs}-\Delta t_{ij}^{cal}\right)}^{2},$$

where $x^{\prime },y^{\prime }\in R$ and $v^{\prime }\in R^{+}$.

**Figure 1:**
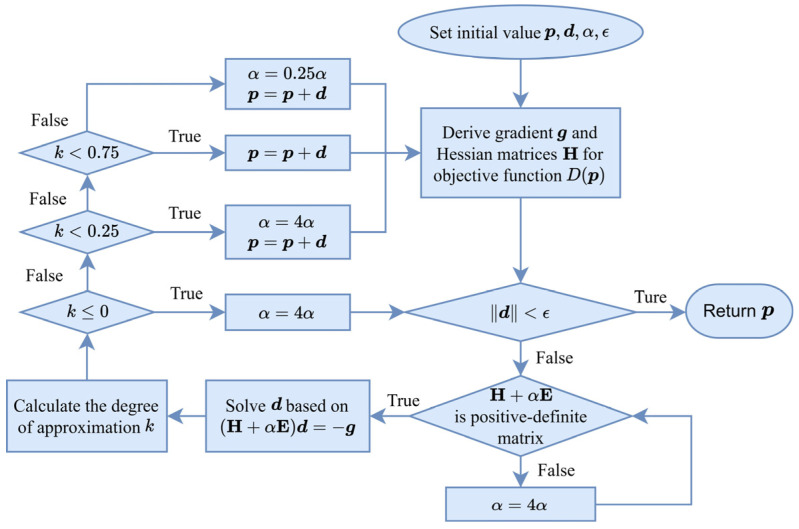
Flowchart of velocity-free localization method.

A unique solution with minimum $D(x^{\prime },y^{\prime },v^{\prime })$ can always be resolved by optimization algorithms. In this work, Levenberg–Marquardt algorithm is used to optimize the objective function and obtain the source position $\left(x_{0},y_{0}\right)$ as well as P-wave velocity v. Figure 1 shows the flowchart of the velocity-free source localization method.

According to Equations (3) and (4), the objective function can be rewritten as

(5) $$D\left(p\right)=\frac{1}{2}f{\left(p\right)}^{T}f\left(p\right),$$

where p is a vector that consists of point coordinates (x,y) and P-wave velocity v. For convenience of calculation, the coefficient 1/2 is added in Equation (5), which does not affect the solution. The function f(p) can be derived from Equation (6). Equation (7) is the Jacobian matrix derived from Equation (6). The gradient g and Hess matrix H of the objective function is then derived by Equation (8) and Equation (9), respectively.

(6) $$f\left(p\right)=\left[\begin{array}{c} f_{1}(p)\\ f_{2}(p)\\ \vdots \\ f_{n}(p) \end{array}\right]=\left[\begin{array}{c} t_{21}^{obs}-\frac{R_{2}-R_{1}}{v}\\ t_{31}^{obs}-\frac{R_{3}-R_{1}}{v}\\ \vdots \\ t_{n1}^{obs}-\frac{R_{n}-R_{1}}{v} \end{array}\right]$$

(7) $$J\left(p\right)=\left[\begin{array}{ccc} \frac{\partial f_{1}\left(p\right)}{\partial x} & \frac{\partial f_{1}\left(p\right)}{\partial y} & \begin{array}{cc} \frac{\partial f_{1}\left(p\right)}{\partial z} & \frac{\partial f_{1}\left(p\right)}{\partial v} \end{array}\\ \frac{\partial f_{2}\left(p\right)}{\partial x} & \frac{\partial f_{1}\left(p\right)}{\partial y} & \begin{array}{cc} \frac{\partial f_{1}\left(p\right)}{\partial z} & \frac{\partial f_{1}\left(p\right)}{\partial v} \end{array}\\ \begin{array}{c} \vdots \\ \frac{\partial f_{n}\left(p\right)}{\partial x} \end{array} & \begin{array}{c} \vdots \\ \frac{\partial f_{n}\left(p\right)}{\partial y} \end{array} & \begin{array}{cc} \begin{array}{c} \vdots \\ \frac{\partial f_{n}\left(p\right)}{\partial z} \end{array} & \begin{array}{c} \vdots \\ \frac{\partial f_{n}\left(p\right)}{\partial v} \end{array} \end{array} \end{array}\right]$$

(8) $$g=\nabla f\left(p\right)=J{\left(p\right)}^{T}f\left(p\right)$$

(9) $$H\approx J{\left(p\right)}^{T}J\left(p\right)$$

The algorithm introduces factor α to ensure that the coefficient matrix H+αE is always positive definite, where E is an identity matrix. Therefore, the search direction d always points in a descent direction. Note that a quadratic function is used to approximate the objective function in this calculation process. To measure the degree of approximation between the quadratic model and the objective function, variable k is derived by

(10) $$k=\frac{{\left\|f\left(p\right)\right\|}^{2}-{\left\|f\left(p+d\right)\right\|}^{2}}{{\left\|f\left(p\right)\right\|}^{2}-{\left\|f\left(p\right)+J\left(p\right)d\right\|}^{2}},$$

where ‖⋅‖ means Euclidean norm. If k is positive (i.e., the search direction does decrease the objective function value), p+d would become a potential value of the real source position and P-wave velocity. According to the value of k, the algorithm would increase, decrease, or keep the value of α. When the length of the search direction is smaller than a given value, for example $\epsilon =1\times 10^{-6}$, it is considered that the algorithm has converged to the optimal point. Therefore, the source position and P-wave velocity are determined.

It is noted that the VFLM does not require a predefined or calibrated wave velocity as an input parameter. In the adopted velocity-free localization framework, the source coordinates and wave velocity can be determined by solving a nonlinear inverse problem based on the arrival-time information. The method assumes that the influence of wave propagation velocity is implicitly contained in the recorded arrival-time information. For relatively homogeneous or weakly heterogeneous media, this assumption can be regarded as an equivalent propagation approximation over the monitored region. However, for strongly heterogeneous or anisotropic materials, path-dependent velocity variations may still affect the arrival-time differences and introduce localization errors. Even in such cases, the method still has a certain velocity-adaptive advantage over conventional velocity-based methods using a constant velocity. The velocity-free source localization program was implemented using Python 3.9.

### 2.2. Location Error Metrics and Objective Function

To evaluate a candidate layout, we compute location errors over a set of grid sources. The location error is defined as the Euclidean distance between the true and estimated source coordinates. Given an error tolerance $e_{tol}$, a localization result is regarded as effective if the error does not exceed $e_{tol}$. The error tolerance should be selected according to the localization accuracy requirement in practical monitoring. In this study, the monitored area was 600 mm × 600 mm. A localization error threshold of 10 mm was adopted for the PLB tests and related sensitivity analyses, corresponding to approximately 1.67% of the characteristic monitoring dimension. This relatively strict threshold was used to provide a more discriminative evaluation of different sensor networks, so that differences in effective monitoring performance could be more clearly identified in the subsequent comparative analyses.

The effective number of location results (NELR) is the total count of effective results over all grid sources, and the effective monitoring range is the corresponding spatial region where effective localization is achieved. NELR represents the number of source points whose localization errors are smaller than $e_{tol}$. Therefore, its absolute value depends on the selected error tolerance and the total number of source points considered in the evaluation. To provide a more intuitive and comparable measure, the NELR percentage is further defined as the proportion of the total monitoring area within the effective monitoring range. Compared with the absolute NELR value, the NELR percentage reduces the influence of the total number of evaluated source points and mainly depends on the $e_{tol}$. In this study, the NELR value, or equivalently the NELR percentage, is used as the primary optimization objective for sensor network design.

### 2.3. Numerical Simulations and Pencil-Lead Break Tests

The numerical simulations were conducted on a 600 mm × 600 mm square plate, where 151 × 151 grid points were obtained by dividing the plate (grid space is 4 mm). The sources were excited sequentially at the grid points. The wave velocity was set as 3000 m/s. The synthetic arrivals were then calculated using Python 3.9 according to Equation (1), where $t_{0}$ was set as 0 s. Table 1 shows the coordinates of the 16 sensors. In this work, the optimal six-sensor network was investigated, so there are $C_{16}^{6}=8008$ sensor network combinations. In each sensor combination, the location results with errors less than $1\times 10^{-10}$ mm are defined as effective location results. The sensor combination with a greater NELR can cover a larger effective monitoring range. It should be clarified that the numerical simulation in this study was conducted under idealized conditions. The theoretical arrival-time information was generated according to the assumed propagation model. No specific source waveform or attenuation curve was assigned, because the main objective of the simulation was to evaluate the influence of sensor layout on localization performance under controlled conditions. A more detailed analysis of the sensitivity to arrival-time noise, velocity uncertainty, and sensor coordinate perturbations will be discussed in Section 3.4.

**Figure 2:**
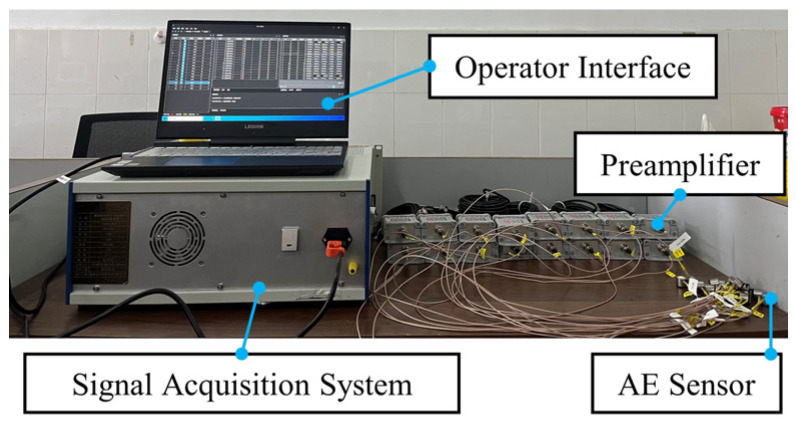
AE sensors, preamplifiers, signal acquisition system, and operator interface of CSZL-1118 multi-channel acoustic emission acquisition system.

**Figure 3:**
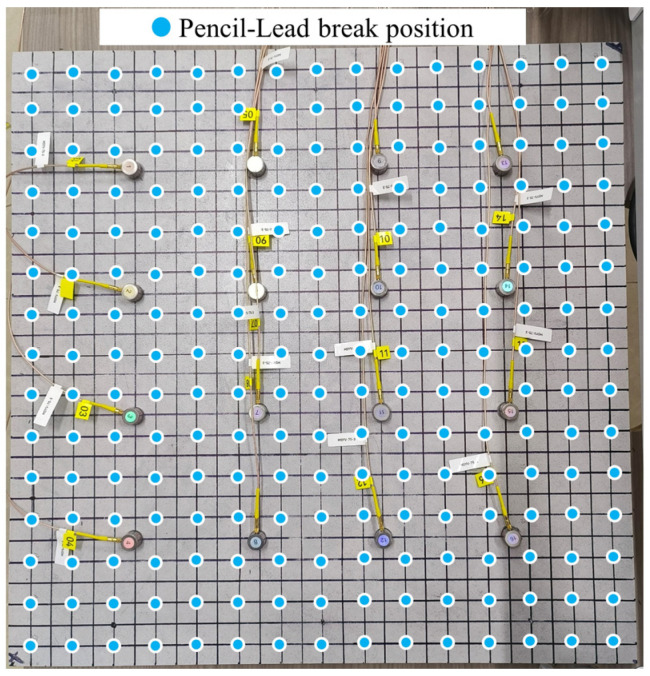
Illustration of the specimen, PLB positions, and sensor positions.

To validate the optimal sensor network derived from numerical simulations, the PLB tests were conducted using a CSZL-1118 multi-channel acoustic emission acquisition system (Hunan Chensheng Zhilin Technology Co., Ltd, Changsha, Hunan, China) (see Figure 2) for real-time AE data acquisition. The system consists of 18 single-channel AE signal processors with 18-bit resolution. The AD-18 sensors, with a diameter of 16 mm, were used to record waveforms. The response frequency ranges from 1 kHz to 500 kHz. The preamplifier, sampling rate, and pre-sampling points were 34 dB, 8 MHz, and 1000, respectively. The threshold value was set to three times the amplitude of the background noise. The specimen is a 600 mm × 600 mm ceramic tile, which is shown in Figure 3. The sensor networks were consistent with numerical simulations. In each sensor combination, location results with errors less than 10 mm are defined as effective location results. To generate AE sources, 225 PLBs were performed in the PLB tests. The pencil lead with a 0.5 mm lead diameter was broken under a 30° contact angle with the plate. The location results using the optimal sensor network were then compared with those using 16 sensors.

## 3. Results and Discussion

### 3.1. Discrete Sensor Network Optimization

Let S be the set of M candidate sensor positions and C⊂S be a selected subset with $\left|C\right|=k$. For each layout C, the velocity-free localization algorithm in Section 2.1 is applied to all test sources, and the corresponding NELR(C) is computed using the error tolerance defined in Section 2.2. The optimal layout is obtained by maximizing NELR(C) over all feasible combinations. In the present case, M=16 and k=6, resulting in 8008 combinations.

### 3.2. Numerical Simulation Results

**Figure 4:**
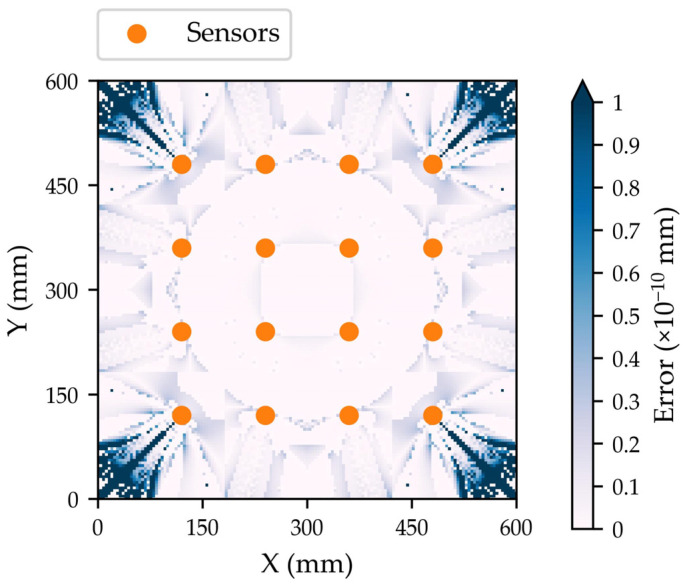
Absolute location errors using 16 sensors in the numerical simulation.

In the numerical simulation, all 16 sensors were first used to locate the AE sources. The location errors are shown in Figure 4. It can be found that the location errors increase in a circular pattern outward from the central region of the sensor network. The location errors of sources in the corner regions are larger than those in other regions. The NELR and NELR percentage of the sensor network in Figure 4 are 21,769 and 95.47%.

**Figure 5:**
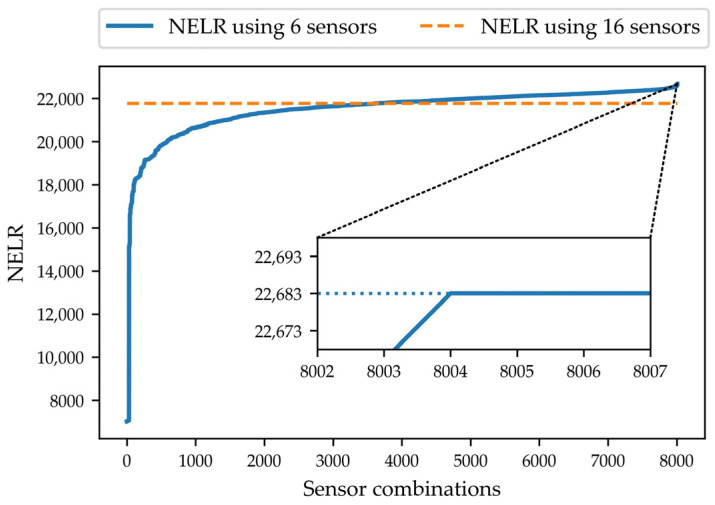
Number of effective location results (NELR) of all six-sensor networks in the numerical simulation.

Secondly, all six-sensor networks were used for AE source localization. The NELR of all six-sensor networks was sorted in ascending order and then plotted into a curve (see Figure 5). Four sensor networks (combination numbers from 8004 to 8007) have the largest NELR, which is 22,683. The corresponding NELR percentage is 99.48%. The sensor networks whose NELR is larger than 21,769 account for 54.8%. These results indicate that sensor layout has an influence on the effective monitoring range, even when the number of sensors is fixed.

**Figure 6:**
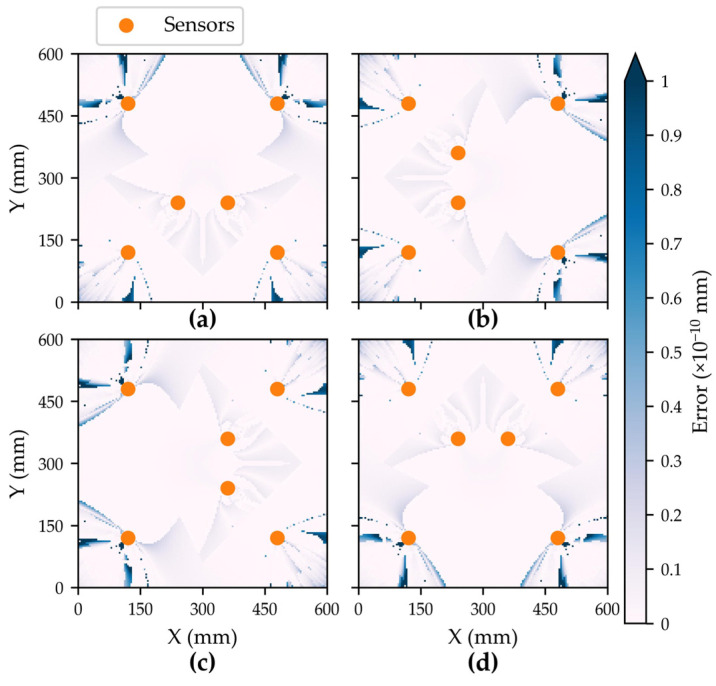
Absolute localization errors of the optimal six-sensor layouts in the numerical simulation. Four sensors are located at the outer corners, while the two remaining sensors are located at (**a**) the bottom inner corners, (**b**) the left inner corners, (**c**) the right inner corners, and (**d**) the top inner corners.

Figure 6 shows the distribution of four optimal sensor networks. In fact, they describe an equivalent sensor network because of the symmetric structure. Compared with the 16-sensor layout in Figure 4, the optimized six-sensor layouts in Figure 6 reduce the localization errors in the corner regions. The four optimal six-sensor networks obtained by the simulation are labeled as FOSNs.

Therefore, the numerical evaluation identifies a family of high-performing layouts sharing a common structural feature: four sensors located at the outer corners of the plate and two sensors placed at inner-corner positions along the horizontal or vertical directions. This configuration provides a larger effective monitoring range, especially in the corner regions outside the sensor network.

### 3.3. Experimental Validation

**Figure 7:**
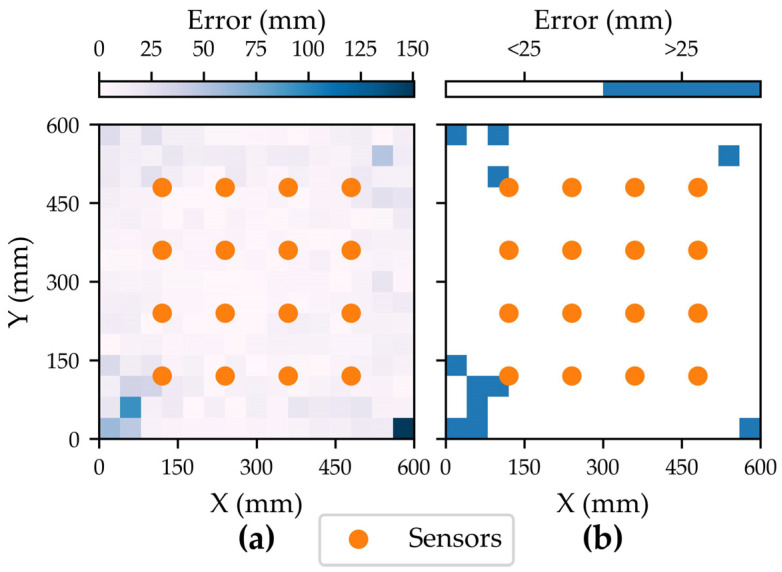
(**a**) Spatial distribution of absolute location errors and (**b**) Spatial distribution of absolute location errors within 25 mm using 16 sensors in the PLB tests.

Similarly, a 16-sensor network was used to determine the PLB sources. From Figure 7a, the main location errors are distributed in the lower-left and lower-right corner regions, which indicates the isotropic structure of the specimen. Although the distribution of location errors does not have obvious symmetry, it is consistent with the distribution in the numerical simulation overall. As shown in Figure 7b, when the PLB positions are close to the center of the sensor network, the location errors range from 0 to 25 mm. While on the outside of the sensor network, especially in corner regions, the location errors are mostly larger than 50 mm, and some even reach 150 mm. Compared with the idealized numerical simulation, the PLB tests include material heterogeneity, anisotropic wave propagation, boundary reflections, sensor coupling conditions, arrival-time picking uncertainty, and possible errors in sensor and source coordinates. These factors can lead to larger localization errors in the experiment.

**Figure 8:**
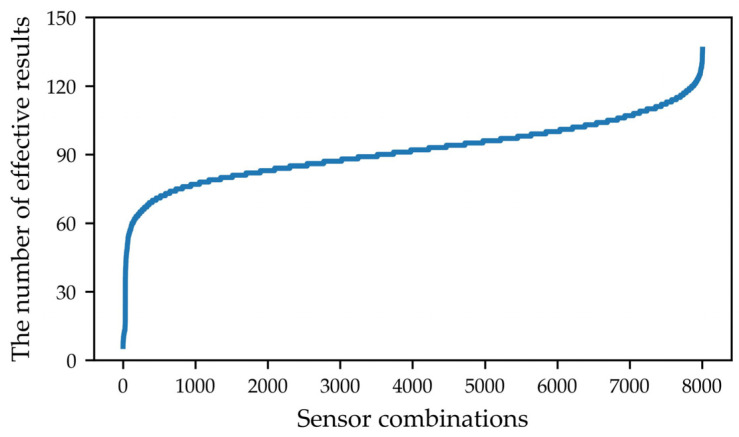
Number of effective results of all six-sensor networks in the PLB tests.

**Figure 9:**
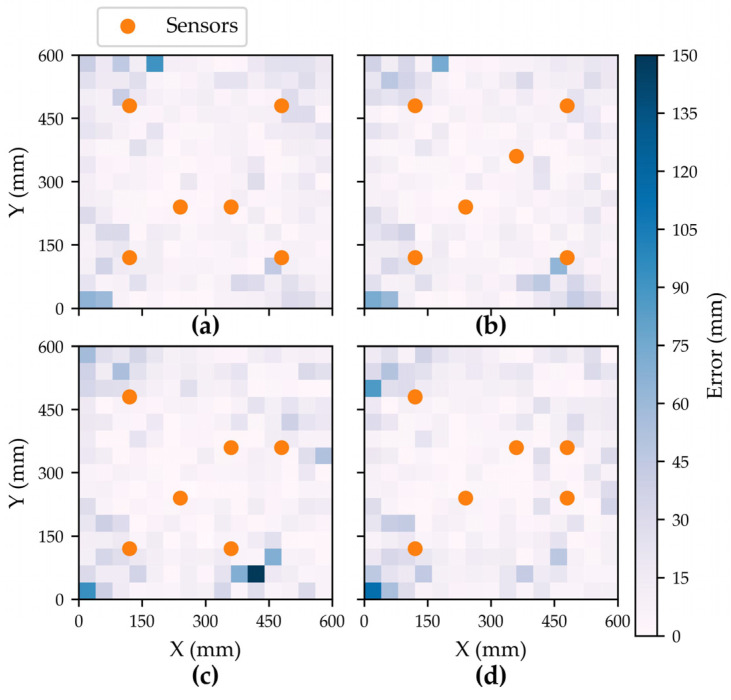
Absolute localization errors of the four best-performing sensor networks in the PLB tests: (**a**) best, (**b**) second-best, (**c**) third-best, and (**d**) fourth-best layouts.

The NELR of all six-sensor networks in the PLB tests is shown in Figure 8. For most sensor networks, the NELR is concentrated around 90. The four optimized sensor networks are shown in Figure 9. The sensor network shown in Figure 9a is the optimal sensor network, with 136 effective location results. It is consistent with the results obtained from the simulation. The sensor networks shown in Figure 9b–d have 135, 134, and 134 effective location results, respectively. Because of anisotropy and inhomogeneity, the location errors in the lower half of the specimen are larger than those in the upper half (see Figure 7). Therefore, the FOSNs are no longer equivalent. To minimize the location errors caused by wave velocity and structural factors in the specimen, the optimal sensor network puts more sensors in the lower half of the specimen. In particular, the sensor networks in Figure 9a,b significantly reduce the location errors in the lower-left and lower-right corner regions.

Therefore, the PLB tests on the same plate specimen confirm the optimal pattern derived from numerical simulations. Layouts with four sensors at the outer corners and two at the inner corners consistently yield a higher proportion of effective localization results under a 10 mm tolerance and reduce corner-region errors compared with alternative six-sensor layouts.

In practical AE monitoring, sensor layout design is affected by both localization accuracy and installation constraints, such as surface accessibility, structural geometry, coupling conditions, and monitoring cost. The proposed framework can help select a reduced sensor set while maintaining the required effective monitoring range, providing a practical balance between monitoring performance and deployment cost. Nevertheless, because the present validation was conducted on a ceramic tile specimen under controlled laboratory conditions, further field validation is needed to assess its applicability to more complex structures.

### 3.4. Comparison with Benchmark and Sensitivity Analysis

#### 3.4.1. Performance of Benchmark

To further evaluate the performance of the proposed method, a comparison with representative existing approaches was conducted. In particular, the optimized sensor network proposed in [39], denoted as SN-FIIEF, was selected as the benchmark. Numerical simulations were performed on a 600 mm × 600 mm plane, with simulated sources arranged at an interval of 4 mm.

**Figure 10:**
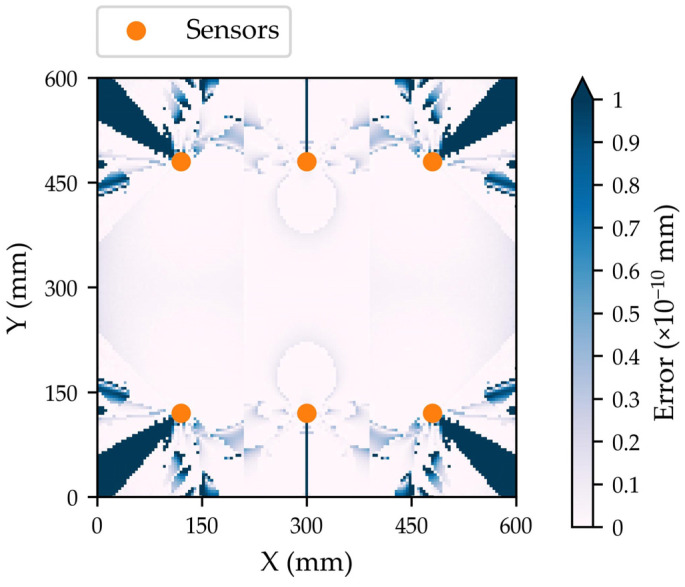
Sensor coordinates of SN-FIIEF and location results under ideal conditions.

As shown in Figure 10, SN-FIIEF provides relatively accurate localization within the sensor network, but its errors increase in the external region, particularly near the four corners of the monitoring area. Its NELR value is 21,052, corresponding to an effective monitoring area percentage of 92.33%. In comparison, the proposed optimized network achieves an NELR value of 22,683 and an effective monitoring area percentage of 99.48%, indicating a larger effective monitoring range, especially in the external region.

#### 3.4.2. Sensitivity to Arrival-Time Noise

To investigate the effective monitoring range of the sensor networks under systematic error and noise disturbance, different levels of random noise were introduced into the theoretical arrival times. The robustness of the SN-FIIEF and FOSN against arrival-pick uncertainty was compared. First, the theoretical arrival times were converted into sample indices according to a preset sampling rate. During this process, rounding errors were introduced, thereby simulating the systematic error of the actual acquisition system. The sample indices were then converted back into arrival times. In this section and in the subsequent numerical tests, the sampling rate was set to 5 MHz.

**Figure 11:**
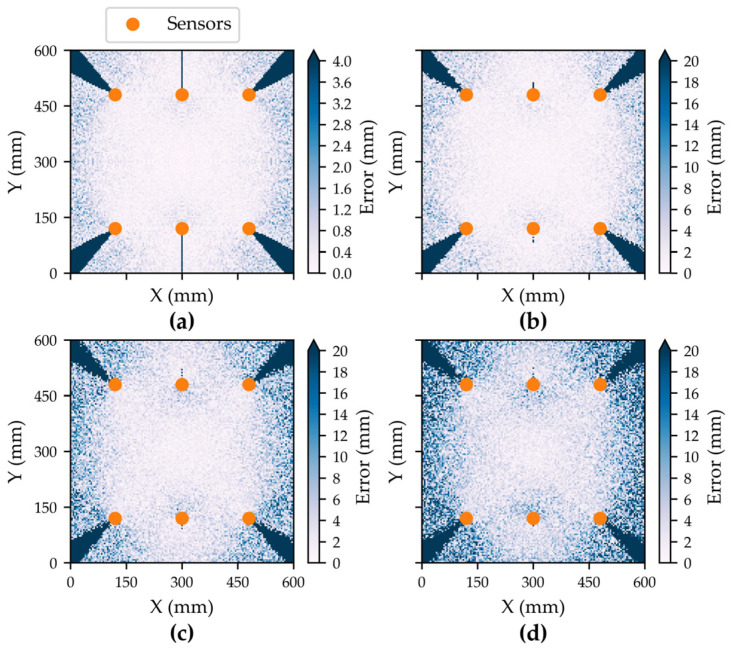
Location errors obtained using the reference sensor network under (**a**) systematic error, (**b**) random arrival-pick errors following U(−2,2) samples, (**c**) random arrival-pick errors following U(−4,4) samples, and (**d**) random arrival-pick errors following U(−6,6) samples.

**Figure 12:**
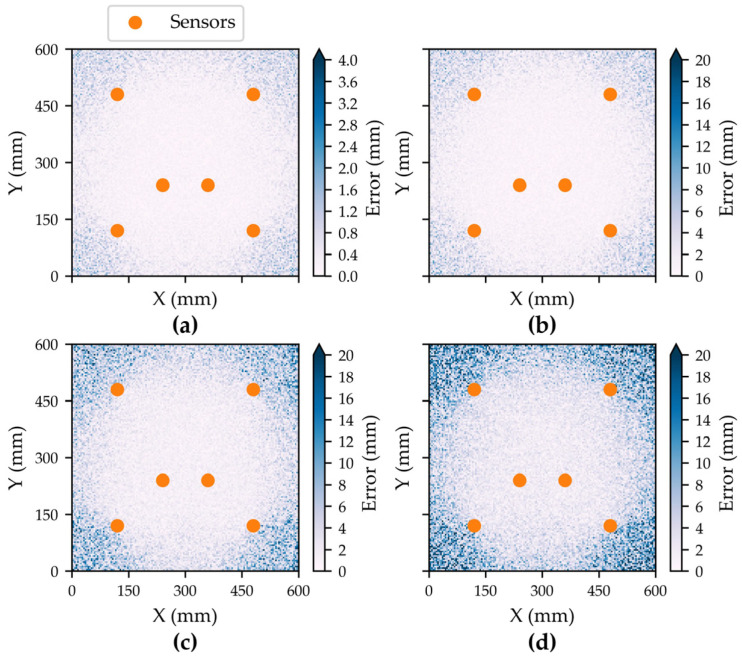
Location errors obtained using the proposed sensor network under (**a**) systematic error, (**b**) random arrival-pick errors following U(−2,2) samples, (**c**) random arrival-pick errors following U(−4,4) samples, and (**d**) random arrival-pick errors following U(−6,6) samples.

To simulate arrival-pick errors under noise disturbance, three levels of uniformly distributed random noise were added to the theoretical arrival picks, namely U(−2,2), U(−4,4), and U(−6,6) samples. Figure 11 and Figure 12 show the monitoring performance of SN-FIIEF and FOSN, respectively. Figure 11a and Figure 12a show the location results considering only systematic error; therefore, the threshold parameter of NELR was set to 2 mm. Figure 11b–d and Figure 12b–d show the location results considering both systematic error and different levels of noise disturbance, for which the threshold parameter was set to 10 mm, consistent with that used in the PLB tests.

Compared with SN-FIIEF, FOSN maintains a larger effective monitoring range under the tested noise levels. The localization errors within the internal region remain relatively stable, and the error increase in the external region is limited. As summarized in Table 2, FOSN achieves a higher NELR percentage than SN-FIIEF under all noise levels, indicating improved robustness against arrival-pick uncertainty.

#### 3.4.3. Sensitivity to Velocity Uncertainty

To investigate the influence of velocity error on the monitoring performance of FOSN, the VFLM and Geiger’s method were used to locate the simulated sources under a noise level of U(−4,4) samples. The true wave velocity in the simulation was 3000 m/s. To introduce different levels of velocity error, the velocity parameter used in the localization algorithms was set to 2000, 2250, 2500, 2700, 2800, 2900, 3000, 3100, 3200, 3300, 3500, 3750, and 4000 m/s. The monitoring performance was then evaluated based on the NELR.

**Figure 13:**
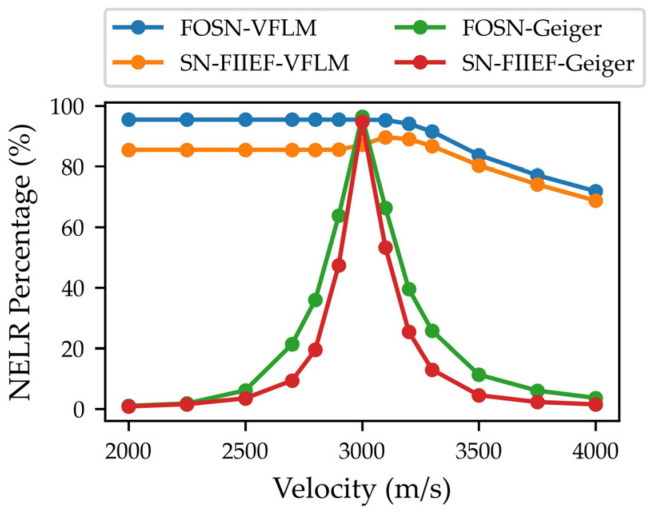
NELR percentages of different combinations of localization algorithms and sensor networks under varying velocity settings. The true velocity is 3000 m/s.

**Figure 14:**
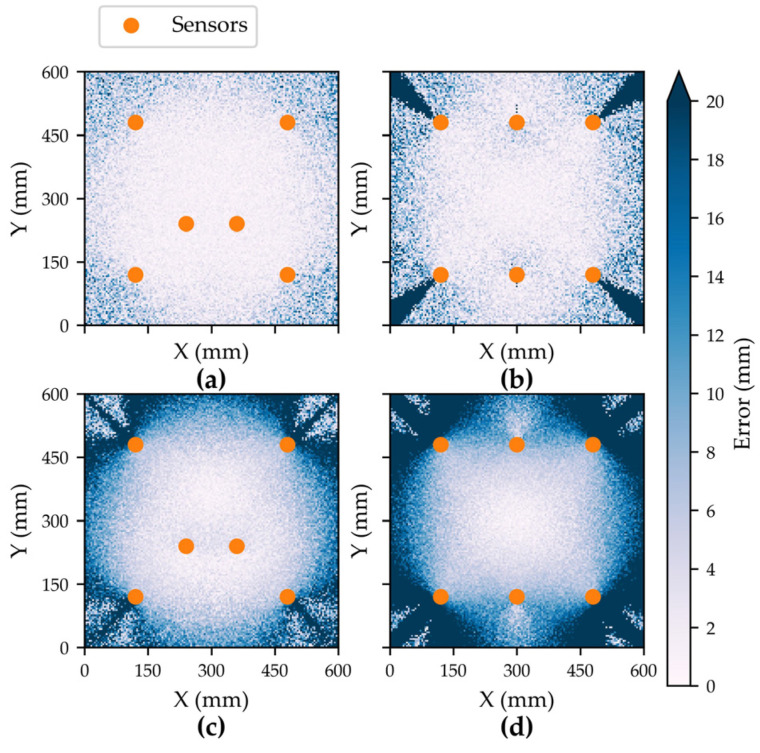
Location results for different combinations of localization algorithms and sensor networks considering velocity error: (**a**) VFLM with FOSN using an initial velocity of 2500 m/s, (**b**) VFLM with SN-FIIEF using an initial velocity of 2500 m/s, (**c**) Geiger’s method with FOSN using an initial velocity of 2900 m/s, and (**d**) Geiger’s method with SN-FIIEF using an initial velocity of 2900 m/s. The true velocity is 3000 m/s.

As shown in Figure 13, under the same localization algorithm and velocity setting, FOSN consistently provides a larger monitoring range than SN-FIIEF. For the same sensor network, VFLM exhibits better stability under different levels of velocity error, whereas Geiger’s method performs well only when the wave velocity is accurately specified. Figure 14 shows the location results for the four combinations. Figure 14a,b show the results obtained using VFLM with an initial velocity of 2500 m/s, whereas Figure 14c,d show the results obtained using Geiger’s method with a velocity setting of 2900 m/s. It can be observed that the combination of FOSN and VFLM achieves the best monitoring performance. In contrast, using either FOSN or VFLM alone may still lead to decreased location accuracy outside the sensor network. These results indicate the improved performance of the proposed method under velocity uncertainty.

#### 3.4.4. Sensitivity to Sensor Coordinate Perturbation

To evaluate the sensitivity of the sensor networks to sensor coordinate perturbation, the monitoring performance of FOSN and SN-FIIEF under different levels of coordinate perturbations was investigated. Based on a fixed arrival-pick error level of U(−4,4) samples, additional sensor coordinate errors of U(−1,1), U(−2,2), U(−3,3), U(−4,4), U(−5,5) mm were introduced. Localization was performed using the VFLM method, and the NELR percentage was adopted as the evaluation metric. For fairness, 50 repeated trials were conducted for each error level, with random seeds ranging from 0 to 49.

**Figure 15:**
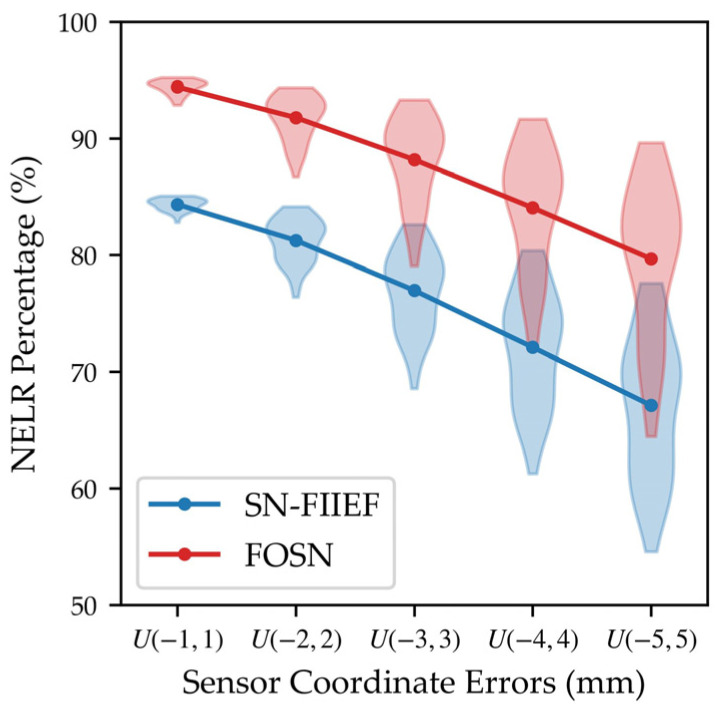
NELR percentages of different sensor networks under varying sensor coordinate errors.

As shown in Figure 15, red and blue denote the results of FOSN and SN-FIIEF, respectively. The shaded regions represent violin plots of the 50 trials, and the dots indicate the mean values. As the coordinate error increases, the effective monitoring ranges of both sensor networks decrease, while the variability of the results becomes more pronounced. When the coordinate error is within ±2 mm, all trials consistently show that FOSN outperforms SN-FIIEF. For larger errors, such as ±5 mm, the NELR distributions of the two networks partially overlap, but the mean NELR percentage of FOSN remains higher across all perturbation levels. These results indicate that FOSN is less sensitive to sensor coordinate errors and maintains a larger effective monitoring range under the tested perturbation conditions.

## 4. Conclusions

In this study, optimal six-sensor networks for velocity-free acoustic emission (AE) source localization on a ceramic tile were investigated through numerical simulations and pencil-lead break (PLB) tests. A velocity-free localization method was employed to determine source positions, and NELR was used to evaluate the effective monitoring range of different sensor layouts. The localization performance of different sensor networks was compared using the NELR metric. In addition, a benchmark comparison was performed with the Fisher-information-based optimized sensor network (SN-FIIEF), and sensitivity analyses were conducted to assess the influence of arrival-time noise, velocity uncertainty, and sensor coordinate perturbations. For the optimal six-sensor networks obtained from the numerical simulations (labeled as FOSN), four sensors were located at the outer corners and the remaining two at the horizontal or vertical inner corners. The optimized sensor network increased the effective monitoring area from 95.47% before optimization to 99.48% after optimization. The PLB tests further confirmed that the optimized six-sensor network improved localization accuracy in regions outside the sensor network. Compared with the benchmark SN-FIIEF, the proposed sensor network achieves a larger effective monitoring range and improved localization performance, especially in external regions. Sensitivity analyses further demonstrate that the optimized sensor network provides enhanced robustness under different levels of arrival-time noise and sensor coordinate errors, maintaining higher effective monitoring ranges and lower variability.

Several limitations should be acknowledged. First, the optimization was performed on a discrete set of candidate sensor positions and for a fixed number of sensors. Second, the numerical simulation was conducted under idealized propagation conditions. Third, the experimental validation was limited to PLB tests on a single ceramic tile specimen under controlled laboratory conditions. Further validation using different materials, structural geometries, and field monitoring conditions will be conducted in future work to assess the broader engineering applicability of the proposed method.

## Figures and Tables

**Table 1:** Coordinates of sensors.

Sensor ID	X/mm	Y/mm
1	120	120
2	240	120
3	360	120
4	480	120
5	120	240
6	240	240
7	360	240
8	480	240
9	120	360
10	240	360
11	360	360
12	480	360
13	120	480
14	240	480
15	360	480
16	480	480

**Table 2:** NELR values and effective monitoring area percentage for different sensor networks under different noise levels.

Noise Level	SN-FIIEF		FOSN	
	NELR	Percentage	NELR	Percentage
systematic error	21,131	92.68%	22,739	99.73%
U(−2,2) samples	21,104	92.56%	22,730	99.69%
U(−4,4) samples	19,489	85.47%	21,762	95.44%
U(−6,6) samples	17,612	77.24%	20,276	88.93%

## Data Availability

The original contributions presented in this study are included in the article. Further inquiries can be directed to the corresponding author.
